# Women With Acute Aortic Dissection Have Higher Prehospital Mortality Than Men

**DOI:** 10.1016/j.jacadv.2023.100623

**Published:** 2023-10-03

**Authors:** Kyohei Marume, Teruo Noguchi, Ryota Kaichi, Takao Yano, Masakazu Matsuyama, Yasuhiro Nagamine, Takayuki Mori, Takafumi Mikami, Sou Ikebe, Masafumi Takae, Soichi Komaki, Masanobu Ishii, Reiko Toida, Kazumasa Kurogi, Yosuke Inoue, Hitoshi Matsuda, Shunsuke Murata, Yuriko Nakaoku, Soshiro Ogata, Kunihiro Nishimura, Takahiro Nakashima, Tetsuro Yamaguchi, Nobuyasu Yamamoto, Kenichi Tsujita

**Affiliations:** a Department of Cardiovascular Medicine, Miyazaki Prefectural Nobeoka Hospital, Miyazaki, Japan; b Department of Cardiovascular Medicine, Graduate School of Medical Sciences, Kumamoto University, Kumamoto, Japan; c Department of Cardiovascular Medicine, National Cerebral and Cardiovascular Center, Suita, Japan; d Department of Critical Care and Emergency Medicine, Miyazaki Prefectural Nobeoka Hospital, Miyazaki, Japan; e Department of Cardiovascular surgery, Miyazaki Prefectural Nobeoka Hospital, Miyazaki, Japan; f Department of Emergency Center, Miyazaki Prefectural Nobeoka Hospital, Miyazaki, Japan; g Department of Cardiovascular Surgery, National Cerebral and Cardiovascular Center, Suita, Japan; h Department of Statistics and Data Analysis, National Cerebral and Cardiovascular Center, Suita, Japan; i Department of Emergency Medicine and Michigan Center for Integrative Research in Critical Care, University of Michigan, Ann Arbor, Michigan, USA; j Department of Internal Medicine, Miyazaki Prefectural Nobeoka Hospital, Miyazaki, Japan

**Keywords:** acute aortic dissection, incidence, prehospital mortality, sex difference

## Abstract

**Background:**

Acute aortic dissection (AAD) often leads to out-of-hospital cardiac arrest (OHCA) and death before hospital arrival.

**Objectives:**

The purpose of this study was to investigate differences in AAD incidence by sex.

**Methods:**

A population-based study in a city with 121,180 residents was conducted using postmortem computed tomography data to identify patients with AAD who died before hospital arrival in 2008-2020. The incidence rate ratio and odds ratio were estimated using Poisson regression and univariable logistic regression, respectively.

**Results:**

A total of 266 patients with incident AAD were enrolled: 84 patients with OHCA, 137 women [n = 137], and 164 patients with type A AAD. The crude and age-adjusted incidence of AAD was 16.2 and 14.3/100,000 person-years, respectively. The incidence of AAD was comparable by sex (men, 16.7/100,000 person-years; women, 15.7/100,000 person-years; incidence rate ratio: 0.94; 95% CI: 0.74-1.20; *P* = 0.64). Compared with men with AAD, women with AAD were older (77 ± 11 years vs 70 ± 14 years; *P* < 0.001), and a higher proportion had type A AAD (76% vs 47%; *P* < 0.001). Women with AAD had higher prehospital mortality than men with AAD (37% vs 21%; *P* = 0.004; OR: 2.24; 95% CI: 1.30-3.87; *P* = 0.004). Among 1,373 patients with OHCA, the proportion of women with AAD was significantly higher than the proportion of men with AAD (11% vs 3.9%; *P* < 0.001; OR: 2.90; 95% CI: 1.86-4.53; *P* < 0.001). AAD was most common in women aged 60 to 69 years (16.4%).

**Conclusions:**

Women had a higher incidence of AAD presenting as prehospital death than men.

Acute aortic dissection (AAD) is a life-threatening condition that can cause sudden death. Therefore, it is challenging to include patients who die before hospital arrival in epidemiological studies of AAD. Several studies from Western countries that have assessed the incidence of AAD included patients who died prehospital using autopsy data (Supplemental Table 1).^1^, ^2^, ^3^, ^4^ However, the autopsy rate in these studies was low. In a study from Iceland, for instance, the autopsy rate declined over time, from 24.0% in 1992 to 9.2% in 2013.^2^ In a recent study from Sweden, the overall autopsy rate was reported as 11%.^3^

Nobeoka City, with a population of 121,180 and 34% of residents aged 65 years or older in 2020,^5^ is isolated from other urban areas. It has only one regional high-quality resuscitation hospital designated by the municipal government, Miyazaki Prefectural Nobeoka Hospital.^6^^,^^7^ In Japan, emergency medical services (EMS) personnel are not permitted to terminate resuscitation in the field. They are instructed to transport patients with out-of-hospital cardiac arrest (OHCA) to the nearest regional high-quality emergency center if cardiopulmonary resuscitation has been initiated. However, EMS personnel do not start cardiopulmonary resuscitation or transfer patients when they identify postmortem rigidity or lividity.^8^ EMS personnel in Nobeoka City transport all patients with OHCA who received cardiopulmonary resuscitation to the Miyazaki Prefectural Nobeoka Hospital (Supplemental Figure 1). At Miyazaki Prefectural Nobeoka Hospital, postmortem computed tomography (PMCT) has been performed since 2008 to identify the cause of death when patients die or are not expected to recover from OHCA as an alternative to autopsy. Generally, PMCT is performed within 2 hours of the pronouncement of death by a physician.

Recently, postmortem imaging was shown to accurately identify the cause of death. Thus, postmortem imaging has emerged as an alternative to autopsy.^9^, ^10^, ^11^ Using PMCT data from Nobeoka City, Yamaguchi et al^7^ investigated the incidence of AAD between 2016 and 2018. They found that the incidence of AAD was 2-fold higher than in previous reports. The aim of this study was to investigate differences in the incidence of AAD by sex among patients with OHCA in a well-defined geographic area.

## Methods

### Study design

All patients with AAD and patients with OHCA in Nobeoka were included using a registry of patients with AAD based on 2 datasets: the AAD dataset for Miyazaki Prefectural Nobeoka Hospital and the OHCA dataset from Nobeoka. The study period was January 2008 to December 2020. This study was conducted in accordance with the Declaration of Helsinki and its amendments. The research ethics committees of Miyazaki Prefectural Nobeoka Hospital (No. 20191004-1) and Kumamoto University (No. 2491) approved this study. As individual patients were not identified, the requirement to obtain informed consent from each study participant or their surviving family members was waived. However, we publicized the study by posting an easy-to-understand summary on the hospital’s website. Participants or their surviving family members were allowed to refuse participation at any time.

### Acute aortic dissection dataset

A database of patients with AAD but not OHCA who visited or were admitted to Miyazaki Prefectural Nobeoka Hospital between January 2008 and December 2020 was generated from the hospital’s medical records based on International Classification of Diseases-10th Revision codes. Patients were extracted using the following key words: aortic dissection, dissecting aortic aneurysm, and ruptured aortic aneurysm. Information on demographics, comorbidities, risk factors, and computed tomography (CT) findings were collected.

### OHCA dataset

Miyazaki Prefectural Nobeoka Hospital has collected the following data on patients with OHCA since 2008: demographic characteristics, initial cardiac rhythm, comorbidities, risk factors, CT findings, and prehospital death. Prehospital death was defined as death in an emergency department after OHCA. Data were reviewed monthly for the cause of death and missing values by a medical committee comprised of physicians, surgeons, emergency physicians, and emergency medical technicians. This committee reviews all OHCAs that involved transport to hospitals in the city. During the study period, 1,373 patients aged ≥18 years who experienced nontraumatic OHCA were transported to hospitals in Nobeoka. Of the 1,373 patients with OHCA, 1,301 (95%) were transported to Miyazaki Prefectural Nobeoka Hospital. PMCT evaluation was performed for 1,235 (90%) patients. The remaining 138 (10%) did not _undergo PMCT evaluation. Of the 138 who did not undergo PMCT evaluation, 52 patients had other obvious causes of OHCA: acute coronary syndrome (n = 19), terminal cancer (n = 10), asphyxia (n = 6), subarachnoid hemorrhage (n = 4), terminal pneumonia (n = 3), idiopathic ventricular fibrillation (n = 2), intoxication (n = 2), aortic stenosis (n = 2), terminal amyotrophic lateral sclerosis (n = 1), Brugada syndrome (n = 1), cerebral hemorrhage (n = 1), and terminal heart failure (n = 1). There were 82 patients who already had a terminal disease; their deaths were classified as natural deaths. The families of 4 patients declined PMCT.

### Diagnosis of AAD

The medical records and CT images of all patients in the OHCA and AAD datasets were reviewed to identify patients with AAD. Miyazaki Prefectural Nobeoka Hospital’s CT and PMCT protocols have been previously described.^7^ Briefly, aortic dissection was diagnosed as a visible intimal flap, inward shift of calcified intima, or high-attenuation crescent in the aorta.^12^, ^13^, ^14^, ^15^ Bloody pericardial effusion was defined as a fluid collection in the pericardial space with 30 to 60 Hounsfield units.^12^^,^^16^ Aortic rupture was diagnosed as periaortic hemorrhage involving the mediastinum, thoracic cavity, or abdominal cavity.^17^ Aortic rupture was classified as aortic dissection if there were clear CT findings of aortic dissection. To identify patients with aortic dissection, CT images of all patients in the OHCA dataset were reviewed by an experienced cardiologist and an experienced radiologist. A blinded third reader adjudicated cases with disagreement. Among 607 randomly selected PMCT evaluations, 11 required a third reviewer due to disagreement between the 2 reviewers. The κ statistic for interobserver agreement on the presence of aortic dissection was 0.87. AAD was distinguished from chronic aortic dissection, defined as occurring <14 days after symptom onset, based on medical history.^18^ AAD was classified into type A, type non-A non-B, or type B. Type non-A non-B AAD was defined as AAD involving the aortic arch and the descending aorta but not the ascending aorta.^19^

### Statistical analysis

Normally distributed continuous variables are expressed as mean ± SD. Categorical variables are expressed as n (%). Groups were compared using Pearson’s chi-squared test for categorical variables and the unpaired t-test for continuous variables. To calculate the incidence of AAD by age group and sex, we divided the number of incident AAD cases by the population of Nobeoka per 100,000. In addition, we performed a direct adjustment to the 2015 Japanese standard population. The incidence rate ratio (IRR) was estimated using Poisson regression. Odds ratios (ORs) were based on univariable logistic regression. Two-tailed *P* < 0.05 was considered statistically significant. We used SPSS version 24.0 (IBM) and Stata 15 (StataCorp) for statistical analyses.

## Results

### Patient characteristics

**Figure 1:**
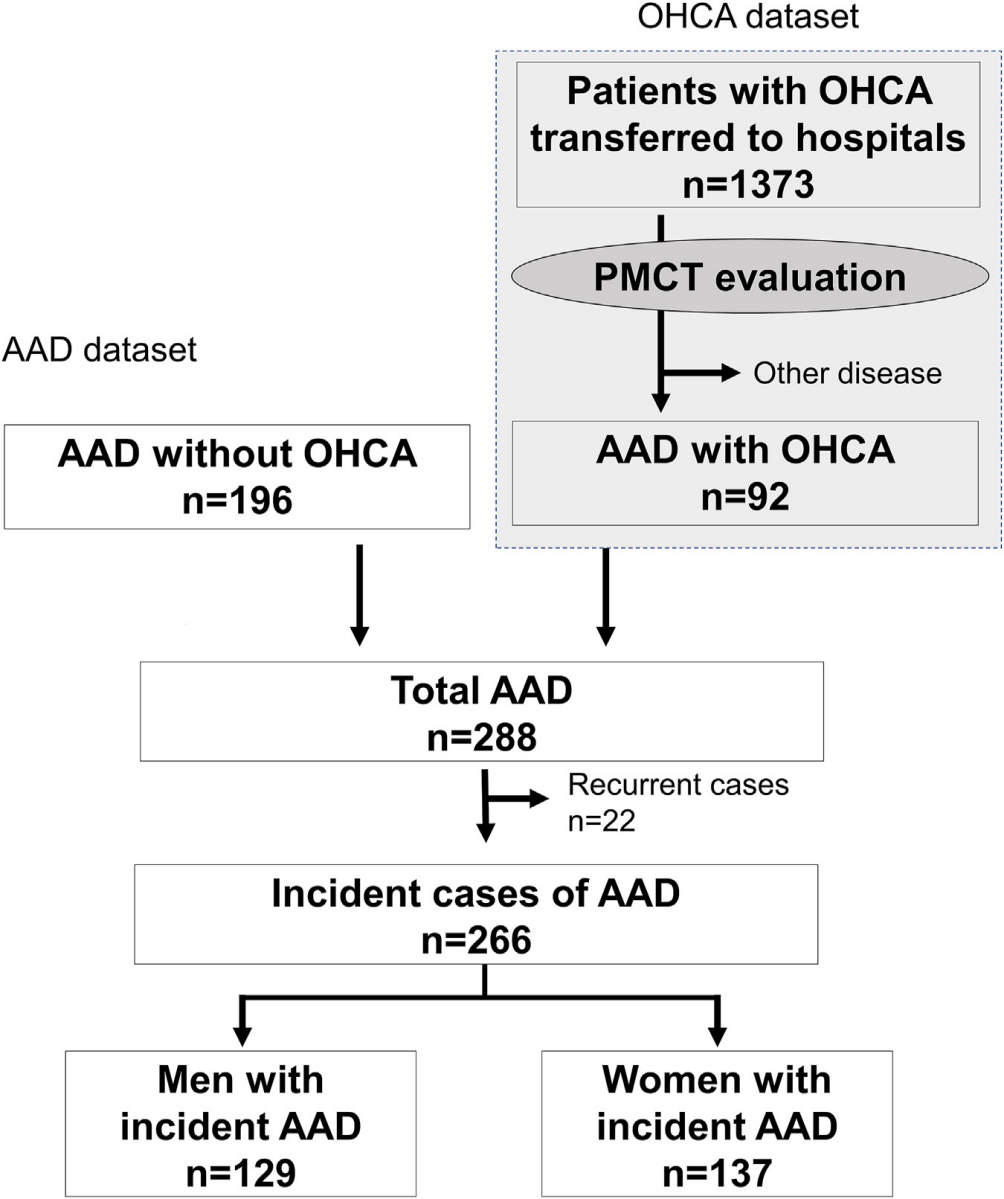
**Study Flow Chart** After excluding 22 recurrent cases, 266 incident cases of AAD were analyzed. There were 84 (32%) patients who experienced OHCA and 164 (62%) patients who were classified as having type A AAD. AAD = acute aortic dissection; OHCA = out-of-hospital cardiac arrest.

A total of 288 AADs were documented. Twenty patients who had recurrent AAD were excluded from this study of incidence. Ultimately, 266 incident AADs were included. There were 137 (52%) female patients. (Figure 1).

Table 1 shows the characteristics of the study patients. The mean age was 74 ± 13 years. There were 164 (62%) patients with type A AAD, 78 (29%) patients with type non-A non-B AAD, and 24 (9%) patients with type B AAD. Women comprised 63% of patients with type A AAD. The most prevalent vascular risk factor for AAD was hypertension. Aortic rupture and bloody pleural effusion occurred in 18% and 38% of patients, respectively. Compared with men with AAD, women with AAD were older (77 ± 11 years vs 70 ± 14 years; *P* < 0.001), and a higher proportion of women had type A AAD (76% vs 47%; *P* < 0.001). The proportion of current smokers among women with AAD was lower than the proportion among men with AAD (6% vs 31%; *P* < 0.001).

**Table 1:** Characteristics of Patients With AAD by Sex (N = 266)

	Overall (N = 266)	Men (n = 129)	Women (n = 137)	*P* Value
Age, y	74 ± 13	70 ± 14	77 ± 11	<0.001
Classification, n (%)				<0.001
Type A	164 (62)	60 (47)	104 (76)	
Type non-A, non-B	78 (29)	57 (44)	21 (15)	
Type B	24 (9)	12 (9)	12 (9)	
Aortic rupture	47 (18)	19 (15)	28 (20)	0.222
Bloody pleural effusion	102 (38)	30 (23)	72 (53)	<0.001
Previous vascular disease (n = 244; n = 116 in men and n = 128 in women)				
Angina	10 (4)	5 (4)	5 (4)	0.874
Acute coronary syndrome	4 (2)	3 (3)	1 (1)	0.268
Stroke	48 (20)	23 (20)	25 (20)	0.954
Peripheral arterial disease	4 (2)	4 (3)	0 (0)	0.035
Risk factor for AAD				
Current smoking (n = 200; n = 101 in men and n = 99 in women)	37 (19)	31 (31)	6 (6)	<0.001
Hypertension (n = 251; n = 122 in men and n = 129 in women)	197 (79)	100 (82)	97 (75)	0.192
Diabetes mellitus (n = 246; n = 117 in men and n = 129 in women)	23 (9)	11 (9)	12 (9)	0.979
Dyslipidemia (n = 244; n = 116 in men and n = 128 in women)	48 (20)	23 (20)	25 (20)	0.954
Heart failure (n = 244; n = 116 in men and n = 128 in women)	30 (12)	16 (14)	14 (11)	0.498
Atrial fibrillation (n = 244; n = 116 in men and n = 128 in women)	18 (7)	5 (4)	13 (10)	0.081
Chronic kidney disease (n = 244; n = 116 in men and n = 128 in women)	41 (17)	28 (24)	13 (10)	0.004
Chronic obstructive pulmonary disease (n = 244; n = 116 in men and n = 128 in women)	10 (4)	8 (７)	2 (2)	0.036
Prehospital mortality	29 (31)	27 (21)	51 (37)	0.004

Values are mean ± SD or n (%).

AAD = acute aortic dissection; OHCA = out-of-hospital cardiac arrest.

### Incidence of AAD

The crude incidence of AAD was 16.2/100,000 person-years. The age-adjusted incidence of AAD for the standard Japanese population in 2015 was 14.3/100,000 person-years. The incidence of type A, type non-A, non-B, and type B AAD was 10.0/100,000 person-years, 4.7/100,000 person-years, and 1.5/100,000 person-years, respectively.

**Figure 2:**
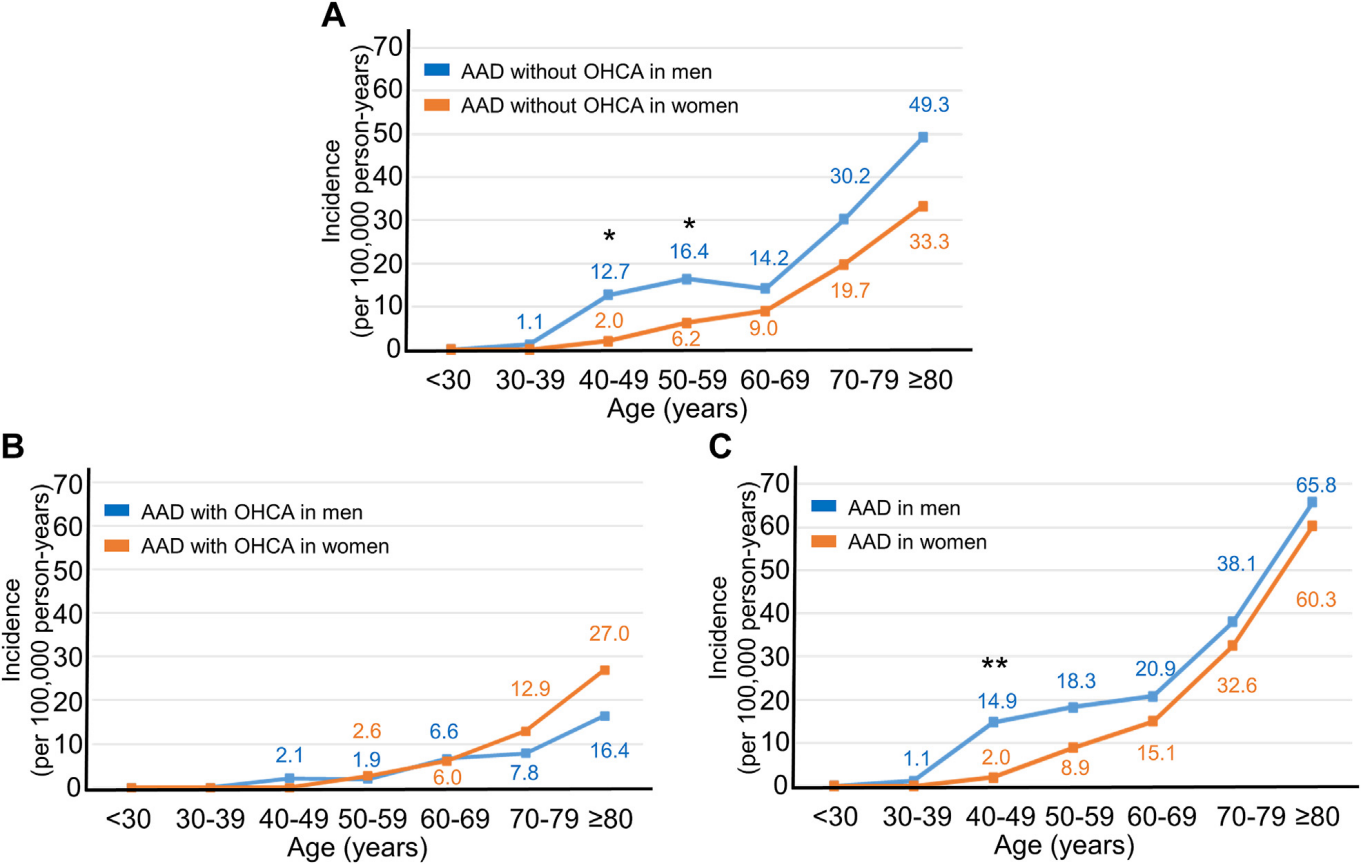
**Incidence of AAD in 10-Year Age Groups Based on the Presence of OHCA** (A) Incidence of AAD without OHCA in 10-year age groups by sex. The incidence of AAD without OHCA is shown in blue for men and orange for women. There was a significant difference in the incidence of AAD without OHCA between men and women in the groups aged 40 to 49 and 50 to 59 years, respectively. ∗*P* < 0.05. (B) Incidence of AAD with OHCA in 10-year age groups by sex. The incidence of AAD with OHCA is shown in blue for men and orange for women. (C) Incidence of AAD in 10-year age groups by sex. The incidence of AAD is shown in blue for men and orange for women. There was a significant difference in the incidence of AAD between men and women in the group aged 40 to 49 years. ∗∗*P* < 0.01.

The incidence of AAD without OHCA was significantly lower in women than in men (men, 13.1/100,000 person-years; women, 9.1/100,000 person-years; IRR: 0.71; 95% CI: 0.53-0.95; *P* = 0.023). This trend was observed in all age groups (Figure 2A). In contrast, the incidence of AAD with OHCA was significantly higher in women than in men (men, 3.6/100,000 person-years; women, 6.4/100,000 person-years; IRR: 1.78; 95% CI: 1.23-2.79; *P* = 0.013). This trend was clear in patients aged 70 to 79 and ≥80 years, respectively (Figure 2B). The overall incidence of AAD in women was comparable to that in men (men, 16.7/100,000 person-years; women, 15.7/100,000 person-years; IRR: 0.94; 95% CI: 0.74-1.20; *P* = 0.64). The incidence of AAD in women was lower in the group aged 40 to 49 years, but women had a similar incidence of AAD as men as age increased (Figure 2C).

### Sex differences in pre-hospital mortality from AAD

Among 266 patients with incident AAD, 84 experienced OHCA. Of 84 patients with AAD and OHCA, 78 died in an emergency department and were diagnosed based on PMCT findings. The prehospital mortality rate was 29% (78 of 266). The prehospital mortality rates of type A, type non-A non-B, and type B AAD were 45% (73 of 164), 4% (3 of 78), and 8% (2 of 24), respectively. Women with AAD had higher prehospital mortality than men with AAD (37% vs 21%; *P* = 0.004; OR: 2.24; 95% CI: 1.30-3.87; *P* = 0.004) (Table 1).

### Proportion of AAD among patients with OHCA

**Figure 3:**
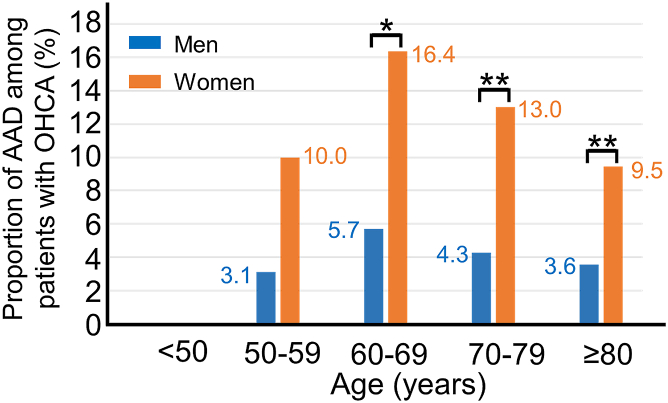
**Age and Sex Differences in Proportion of AAD Among Patients With OHCA** The blue bar shows the proportion of men with OHCA who had AAD. The orange bar shows the proportion of women with OHCA who had AAD. There was a significant difference by sex in the groups aged 60 to 69, 70 to 79, and ≥80 years. ∗*P* < 0.05, ∗∗*P* < 0.01.

Among 1,373 patients with OHCA, 92 (6.7%) had AAD. This included 8 (9%) patients with recurrent AAD and 84 (91%) with type A AAD. The most common initial cardiac rhythm of patients with AAD was pulseless electrical activity (n = 43, 47%). Ventricular fibrillation (n = 2, 2%) and pulseless ventricular tachycardia (n = 0, 0%) were rare (Table 2). Among patients with OHCA, a higher proportion of women had AAD than men (11% vs 3.9%; *P* < 0.001; OR: 2.90; 95% CI: 1.86-4.53; *P* < 0.001). The interaction between age and sex was not significantly associated with the proportion of AAD among patients with OHCA (*P* = 0.50). By age group, women aged 60 to 69, 70 to 79, or 80 to 89 years were more likely to have AAD than men in the same age groups (age 60-69 years: OR: 3.23; 95% CI: 1.18-8,86; *P* = 0.023; age 70-79 years: OR: 3.36; 95% CI: 1.44-7.85; *P* = 0.005; age 80-89 years: OR: 4.85; 95% CI: 1.94-12.13; *P* = 0.001). Among women aged 60 to 69 years, 16.4% had AAD, which was the highest percentage (Figure 3).

**Table 2:** Characteristics of Patients With OHCA Transported to a Hospital (N = 1,373)

	All Transported Patients (N = 1,373)	Patients With AAD (n = 92)
Age, y	77 ± 14	78 ± 10
Female	579 (42)	61 (66)
Initial cardiac rhythm		
Ventricular fibrillation	81 (6)	2 (2)
Ventricular tachycardia	38 (3)	0
Asystole	867 (63)	46 (50)
Pulseless electrical activity	359 (26)	43 (47)
Unknown	28 (2)	1 (1)

Values are mean ± SD or n (%).

AAD = acute aortic dissection; OHCA = out-of-hospital cardiac arrest.

## Discussion

This study is the first to show that AAD is more common among women than men with OHCA. Women had a higher incidence of AAD presenting as sudden death than men. One-third of women with AAD died before hospital arrival (Central Illustration).

**Central Illustration:**
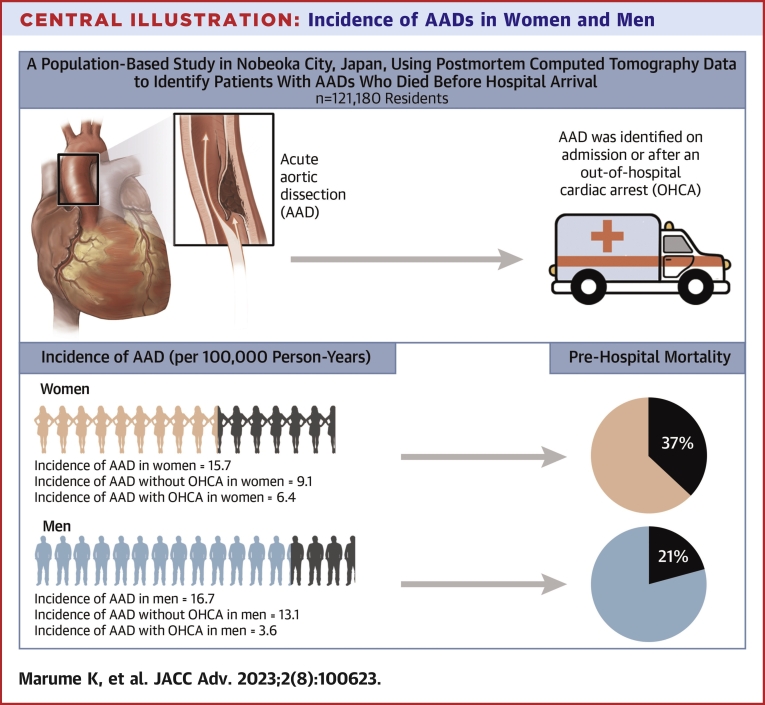
**Incidence of AADs in Women and Men** A population-based study in a city with a population of 121,180 used postmortem computed tomography data from out-of-hospital arrests over 13 years (2008-2020) to investigate differences in the incidence of AAD by sex. The overall incidence of AAD in women and men was comparable (men, 16.7/100,000 person-years; women, 15.7/100). The incidence of AAD without OHCA was significantly lower in women than in men (men, 13.1/100,000 person-years; women, 9.1/100,000 person-years). In contrast, the incidence of AAD with OHCA was significantly higher in women than in men (men, 3.6/100,000 person-years; women, 6.4/100,000 person-years). Women with AAD had higher prehospital mortality than men with AAD (37% vs 21%). AAD = acute aortic dissection.

### Sex differences in the incidence and characteristics of AAD

The International Registry of Acute Aortic Dissection enrolled 1,078 patients with AAD. In that registry, 32% of women had AAD,^20^ which was cited in recent guidelines as a sex difference in AAD incidence.^21^^,^^22^ Similarly, the Spanish Registry of Acute Aortic Syndrome showed a lower proportion of patients with AAD were women (27%).^23^ However, these studies did not enroll patients who died before hospital admission. Population-based studies of AAD using autopsy data to enroll patients who died before hospital admission have shown a higher proportion of women (36%-40%) with AAD^1^, ^2^, ^3^, ^4^ than the registries mentioned above, but the incidence of AAD was lower in women than in men,^2^, ^3^, ^4^ In the present study using PMCT data, 52% of patients with AAD were women, and the incidence of AAD was comparable between men and women. Women with AAD had higher prehospital mortality in this study. A previous nationwide population-based study showed higher 30-day mortality in female patients admitted with aortic dissection than male patients admitted with aortic dissection and that the proportions of women were higher among patients deceased without hospital admission than in hospitalized patients. The authors of that study suggested the higher mean age of women might have contributed to differences by sex.^3^ A higher short-term mortality rate in women compared to men has also been reported in patients with acute myocardial infarction. Women with acute myocardial infarction are typically older and have more comorbidities.^24^ These findings might be helpful in assessing the reason for sex-specific mortality differences in AAD. In our study, women with AAD were older and less likely to be current smokers than men with AAD. The prevalence of hypertension was comparable. Notably, the higher proportion of women with type A AAD might have strongly contributed to higher mortality in women. Further studies are needed to assess the reasons for sex-specific mortality differences in AAD. The incidence of AAD in women could have been previously underestimated because more than one-third of women with AAD died before hospital arrival.

The incidence of AAD in the present study might have been affected by the higher age of this study population relative to other study populations because age is a risk factor for AAD. By including a substantial number of patients with AAD and OHCA identified by PMCT, we found that the incidence of AAD was lower in women in younger age groups, but the incidence in women caught up with the incidence in men as age increased (Figure 2). This suggests that the incidence of AAD in women and men will be comparable in other areas with aging populations. Our results might be generalizable for Japan because our study population distribution is similar to that of Japan as a whole. Our results can help predict the trajectory of other countries with aging populations. These results help inform future studies aimed at better understanding the biology of AAD and AAD prevention and treatment strategies throughout the world.

Prehospital mortality in this study (29%) was similar to mortality before hospital arrival or admission from previous reports based on autopsy data (17.6%-38%).^1^^,^^3^^,^^4^ Considering the low autopsy rates in other studies, mortality due to AAD could have been underestimated. Population-based studies in other countries with a higher autopsy rate or studies with PMCT data are warranted for comparisons of AAD incidence and mortality with those found in the present study.

### Sex differences in the proportion of patients with OHCA and AAD

This study is the first to show that AAD is more common among women with OHCA than men with OHCA. The proportion of patients with OHCA who have AAD was recently investigated in other Japanese studies. Two recent studies evaluated approximately 90% of all consecutive patients with OHCA who were transported to hospitals. They showed that 7.6% of patients with OHCA who underwent CT examination had AAD; 7.0% to 7.1% had type A AAD.^12^^,^^25^ We showed a similar proportion of patients with OHCA had AAD (6.7%) and type A AAD (6.1%) in this study, which evaluated 90% (1,235/1,373) of transported patients with OHCA in a city with a population of 120,000. It should be noted that a substantial proportion of patients with OHCA aged 50 to 59 years or 60 to 69 years had AAD; the highest proportion was 16.4% in women aged 60 to 69 years. To develop a practical treatment strategy, it is important to strongly consider AAD in women with OHCA.

### Study Limitations

First, autopsies were not performed in all cases. Although the diagnostic value of PMCT for AAD is high, there are limitations in diagnosing aortic dissection based on CT, especially noncontrast PMCT. In the present study, AAD was diagnosed based on the presence of clear CT findings. There is a possibility that patients with no clear findings of AAD on CT were not included. For instance, in patients with aortic rupture, diagnosis of AAD based on PMCT was particularly difficult. Second, not all individuals who die in Nobeoka City undergo PMCT. For example, family members of patients with terminal diseases who did not want to be resuscitated did not call EMS or transport them to hospitals. In addition, if postmortem rigidity or lividity was evident, EMS personnel did not initiate resuscitation or transport the patient to a hospital. During the study period, 1,096 people with nontraumatic OHCA in Nobeoka City were not transported to hospitals because of apparent postmortem rigidity or lividity. Among 1,373 patients with OHCA who were transported to hospitals, 138 (10%) did not undergo PMCT evaluation. Of these, 52 (38%) patients had an obvious cause of OHCA. The remaining 86 (62%) patients could have had AAD. In the field of epidemiological studies of AAD, there is a need to continue evaluating more prehospital deaths in order to increase the accuracy of epidemiological information. Third, the incidence of AAD observed in our study might be a reflection of unidentified genetic factors, environmental factors, or both. Japanese people have the longest life expectancy in the world.^26^ Moreover, climate can affect the incidence of AAD.^27^ However, comprehensive information from Japan, which has a rapidly aging population, can be crucial for developing perspectives in other countries. Fourth, for patients with OHCA, it was difficult to obtain the medical history and other information on background factors during the course of medical treatment. Therefore, it was not possible to adjust for factors such as chronic kidney disease or chronic obstructive pulmonary disease when calculating odds ratios for the presence of AAD in patients with OHCA.

## Conclusions

Women have a comparable incidence of AAD as men, but they have higher prehospital mortality. A higher proportion of women with OHCA had AAD than men with OHCA, with the highest proportion in women aged 60 to 69 years, at 16.4%.

PERSPECTIVES **COMPETENCY IN MEDICAL KNOWLEDGE:** The incidence of AAD could have been previously underestimated in women because they die before hospital arrival at a higher rate than men. It is important to strongly consider AAD for women with OHCA.

**TRANSLATIONAL OUTLOOK:** In the field of epidemiological studies of AAD, there is a need to continue evaluating more prehospital deaths in order to increase the accuracy of epidemiological information.

## Funding support and author disclosures

This work was supported in part by the 10.13039/100007449Takeda Science Foundation, the 10.13039/100015639Japan Cardiovascular Research Foundation, and 10.13039/501100001691JSPS KAKENHI Grant Number 22K16077. The authors have reported that they have no relationships relevant to the contents of this paper to disclose.
