# Supplementary material for: Women With Acute Aortic Dissection Have Higher Prehospital Mortality Than Men
Source: JACC Adv. 2023 Oct 3;2(8):100623. doi: 10.1016/j.jacadv.2023.100623 (PMC11198350; doi:10.1016/j.jacadv.2023.100623)

**Supplemental Table 1. Comparison of study methodologies between previous studies and this study**

|  | | | | **Evaluation of patients who died before hospital admission** | |  |  |
| --- | --- | --- | --- | --- | --- | --- | --- |
| **Authors** | **Study population (location)** | **Study period** | **Incidence (/100,000 person-years)** | **Modality** | **Evaluation rate** | **Limitations** |  |
|  |  |  |  |  |  |  |  |
| **Excluded patients with OHCA** | | | | | | |  |
| McClure et al ^16^ | 13.5 million (Ontario, Canada) | 2002–2014 | Thoracic aortic dissection, 4.6 | NA | NA | No evaluation of patients who died before hospital admission |  |
| **Included patients with OHCA** | | | | | | |  |
| Clouse et al ^4^ | 100,000 (Olmsted County, US) | 1980–1994 | AAD, 3.5 | Autopsy | Not mentioned | Unknown autopsy rate |  |
| Melvinsdottir et al ^2^ | 290,072 (Iceland) | 1992–2013 | Thoracic AAD, 2.5 (Men, 3.2; Women, 1.9) | Autopsy | 15.40% | Autopsy rate was 15.4% |  |
| Smedberg et al ^3^ | 10 million (Sweden) | 2002–2016 | Aortic dissection, 7.2 (Men, 9.1; Women, 5.4) | Autopsy | 11.00% | Autopsy rate was 11.0% |  |
| Howard et al ^1^ | 92,728 (Oxfordshire, UK) | 2002–2012 | AAD, 6.0 (Men, 4.9; Women, 3.7) | Autopsy | Not mentioned | Unknown autopsy rate |  |
| Yamaguchi et al ^8^ | 120,000 (Nobeoka, Japan) | 2016–2018 | AAD, 17.6 (Men, 22,3; Women 15.9) | PMCT | 93% of transported patients with OHCA | Short study period Included recurrent cases No evaluation of non-transported patients with OHCA |  |
| Marume et al (this study) | 120,000 (Nobeoka, Japan) | 2008–2020 | AAD, 16.2 (Men, 16.7; Women, 15.7) | PMCT | 90% of transported patients with OHCA | No evaluation of non-transported patients with OHCA |  |

AAD, acute aortic dissection; OHCA, out-of-hospital cardiac arrest, PMCT, postmortem computed tomography.

Supplemental Figure 1


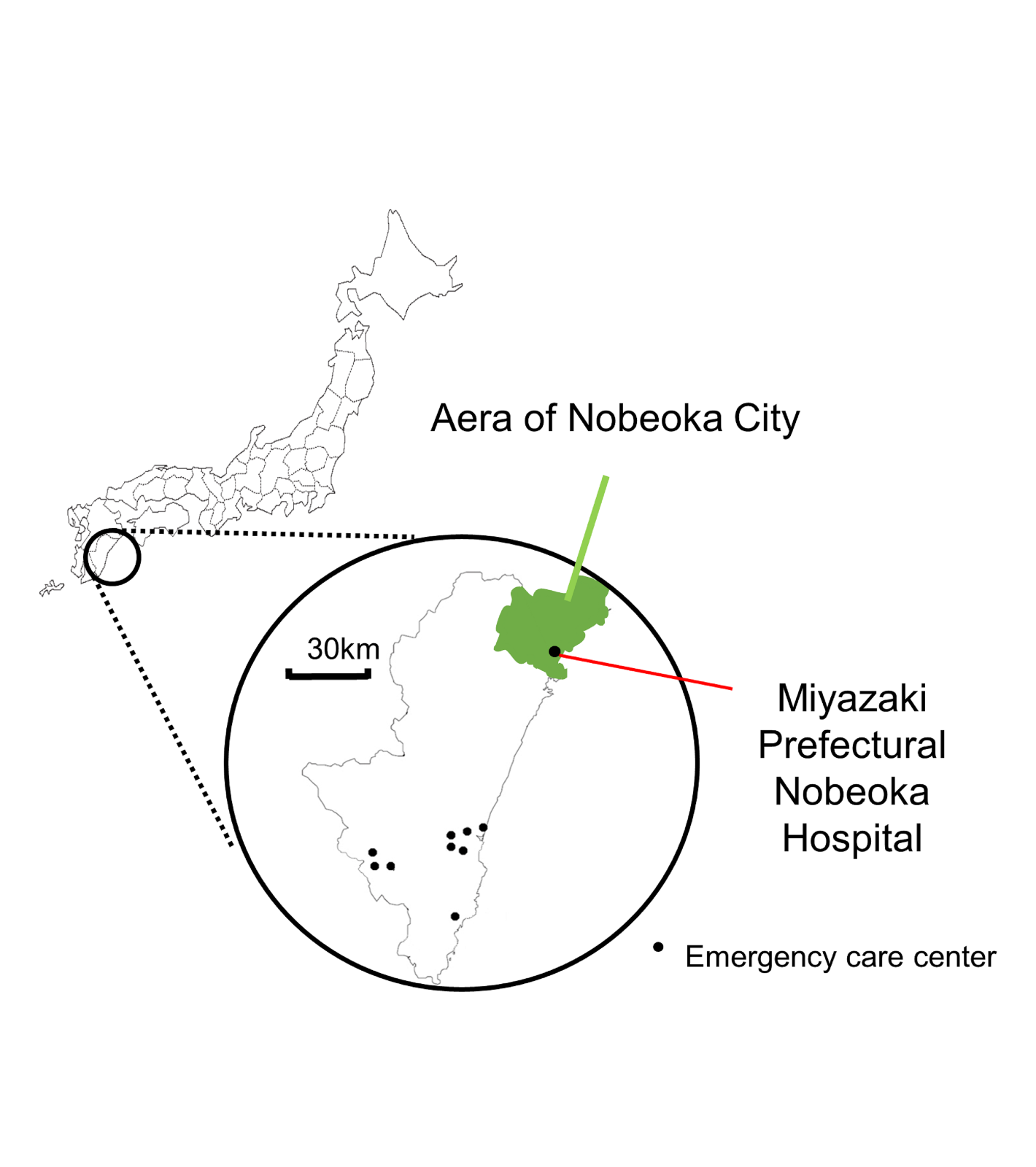

Supplement: Supplemental Figure 1 and Supplemental Table 1 [file mmc1.docx]
